# Formic and propionic acids' effectiveness on laying hens' productivity and egg quality, utilization of nutrients and some blood profiles during the early production phase

**DOI:** 10.1007/s11250-025-04276-z

**Published:** 2025-02-15

**Authors:** Fayza M. Salem, A. A. Abd El-Dayem

**Affiliations:** Animal and Poultry Nutrition Department, Desert Research Center, Cairo, Egypt

**Keywords:** Laying hens, Propionic acid, Formic acid, Early-phase egg production

## Abstract

The use of organic acids as growth promoters in poultry diets continues to pique the researchers’ curiosity. So, this study aims to investigate the effect of feeding formic acid, propionic acid, or both in their pure form on laying hens' productivity, egg quality, nutrient utilization, and some blood profiles during the early production phase using eighty ISA Brown laying hens aged 28–42 weeks. The hens were randomly assigned into four groups, each consisting of 10 replicates with 2 hens/replicate. The treatments were C (control), F (1 ml formic acid/kg diet), P (1 ml propionic acid/kg), and M (0.5 ml formic acid and 0.5 ml propionic acid/kg). The percentage of egg production, egg mass (*P* < 0.05), albumen crude protein percent, and estradiol hormone (*P* < 0.001) were significantly increased upon acids’ mixture treatment, whereas the percentages of albumen fat (*P* < 0.05) and yolk fat (*P* = 0.0006) were significantly decreased. Formic acid significantly increased the percentages of shell calcium (*P* = 0.0001) and yolk (*P* < 0.01) and decreased albumen percent (*P* < 0.01). The acids’ mixture produced the lowest plasma cholesterol value (*P* = 0.0001), which was followed by formic acid treatment. Unlike the control, the experimental treatments significantly increased Haugh unit, albumen index (*P* < 0.05), crude protein retention percent (*P* < 0.01), and decreased plasma triglycerides (*P* = 0.0007). According to the findings, early-phase laying hens' diets supplemented with a pure blend of propionic and formic acids produced more eggs and had higher egg quality.

## Introduction

There is still considerable attention and research being done on organic acids as feed acidifiers for poultry diets. Organic acids come in a variety of forms, including formic, propionic, lactic, citric, acetic, fumaric, and others. Each type of acid acts differently and in different sectors or locations. In poultry nutrition, formic and propionic acids are utilized as feed acidifiers. Acidifiers are included in poultry diets to lower the pH of the gut, thereby limiting the growth of bacteria less tolerant to acid pH (Hernández et al. 2006). Gut health is regarded as an important factor influencing birds productivity, and thus the economics and profit of the poultry industry. Organic acids can change the pH of the intestinal tract, which enhances the solubility, digestion, and nutrient absorption of the feed components (Adams 1999), as a result, chicken productivity is increased. Propionic acid has a potent bacteriostatic and mold-inhibiting effect. It effectively prevents digestive disorders in poultry. The pathogenic bacterial cell can no longer divide or may even die consequently of propionic acid's ability to break down the DNA structure in its nucleus (Haque et al. 2009). These bacteria are sensitive to low pH, but beneficial bacteria are unaffected (Haque et al. 2009). The addition of propionic acid to poultry feed lowers the gut’ pH, inhibiting bacterial growth while improving performance (Haque et al. 2009). Additionally, formic acid is used as an antimicrobial to limit feed pathogen growth and their subsequent establishment in the poultry intestine. Formic acid seems to be antagonistic to pathogens once it is ingested with feed or in drinking water. According to earlier research, adding an encapsulated organic acids mixture to a layer diet increased the treated groups' feed intake, feed conversion, and egg production when compared to the untreated group (Youssef et al. 2013). Similarly, adding an organic acids blend with 40% formic acid to the diet of laying hens improved the feed conversion ratio and increased average egg weight and production capacity during the trial period (Grashorn et al. 2013). Furthermore, laying hens fed a combination of propionic and formic acids showed a significant increase in both egg production and egg mass versus the control during the last stage of production (Salem 2020). On the other hand, a blend of organic acids (benzoic acid, fumaric acid, phosphoric acid, and formic acid) at 2 or 3 g/kg diet for laying ducks significantly increased daily feed intake, Haugh unit, and yolk weight compared to the control (Cao et al. 2022). However, unlike their salts, free formic acid and propionic acid have not been widely used as dietary supplements due to their pungent smell. So, this study aims to investigate the effect of feeding formic acid, propionic acid, or both in their pure form at low levels on laying hens' productivity, egg quality, nutrient utilization, and some blood profiles while having no negative impacts on hen health during the early production phase using eighty ISA Brown laying hens aged 28–42 weeks.

## Materials and methods

### The experimental design and bird housing

This research took place at the South Sinai Experimental Research Station (Ras-Suder City), which is part of the Desert Research Center in Cairo, Egypt. Eighty ISA Brown laying hens aged 28–42 weeks were randomly distributed into 4 experimental groups; each consisting of 10 replicates with 2 hens/replicate. The treatments of this study were control treatment, C (basal diet), F (basal diet plus 1 ml formic acid/kg diet), P (basal diet plus 1 ml propionic acid/kg diet), and M (basal diet plus 0.5 ml formic acid and 0.5 ml propionic acid/kg diet). The hens were raised in wire cages within triple-deck batteries, with identical environmental and hygienic conditions, and 16 h of continuous light. An electronic digital thermo-hygrometer was used to measure the average indoor ambient temperature (20.86ºC ± 0.27) and relative humidity (57.94 RH (%) ± 1.05). The diet formula is shown in Table 1 NRC (1994). The diet was iso-caloric (2677.6 kcal ME/Kg) and iso-nitrogenous (18.03% CP). Feed and water were available ad libitum.

**Table 1:** The composition of the experimental diet

Ingredients	%
Yellow corn	58.40
Soybean meal 44%	20.50
Corn gluten meal 60%	5.00
Limestone	7.50
Di-Calcium phosphate	1.70
Wheat bran	6.00
DL-Methionine	0.30
Salt	0.30
Vit&Min. Premix*	0.30
Total	100
Calculated Values	
CP %	18.03
ME (Kcal/kg)	2677.6
Ca %	3.30
Available P %	0.43
DL-Methionine %	0.62
L-Lysine %	0.79

*Vitamins and minerals premix, each 2.5 kg contain: Vit A 10000000 IU, Vit D3 2000000 IU, Vit E 10 g, Vit K 1000mg, Vit B12 10mg, Vit B1 1000mg, Vit B2 5000mg, Vit B6 1.5g, Pantothenic acid 10g, Niacin 30g, Folic acid 1g, Biotin 50mg, Iron 30g, Manganese 70g, Choline chloride 10g, Copper 4g, Zinc 50g, Selenium 100mg and Iodine 300mg

### Experimental measurements

Feed consumption was recorded weekly. Egg mass (g/hen/day) was calculated using daily egg number and weight data. The amount of feed consumed divided by the mass of the egg is known as the feed conversion ratio (g feed intake/g egg mass). Ten eggs were collected from each treatment at the conclusion of the experiment to measure the quality attributes of the eggs and shell, such as the egg's weight, shape index, albumen% and index, yolk% and index, Haugh unit, shell weight, shell%, thickness, density, shell Ca%, shell surface area, and shell weight per unit of surface area (SWUSA). The weights of the albumen, yolk, and shell percentages were determined in relation to the egg weight (Carter 1968). The following formula was used to calculate the egg-shape index (Panda 1996):

$$Egg\;shape\;index = (Egg\;width\;/\;Egg\;length) * 100$$

The following equation was used to calculate the Haugh unit according to Haugh (1937):

$$Haugh\;unit = 100 \times log\;(H+7.57-1.7\times {W}^{0.37})$$

where: H = Albumen height, W = Egg weight

Shell thickness (ST) was measured with a micrometer, and shell weight per unit of surface area (SWUSA) and shell surface area (SA) were computed using the following formulas (Nordstrom and Qusterhout 1982):

$$SA\;({cm}^{2}) = 3.9782 \times {EW}^{0.7056}$$

$$SWUSA\;(mg/{cm}^{2}) = SW \;(mg)\;/SA\;({cm}^{2})$$

where: 3.9782 = constant factor, EW = egg weight (g), SW = shell weight (g).

The shell density (SD) in g/cm3 was calculated using the following equation (Curtis et al. 1985):

$$SD = SW\;(g)/SA\;({cm}^{2}) \times ST\;(cm)$$

Shell Ca% was determined by the Ion Conductively Plasma (ICP) ASTM (2002).

### The chemical composition of egg yolk and albumen

The chemical composition of egg yolk and albumen was determined by using 5 eggs from each treatment according to A.O.A.C. (2005).

### Digestibility trial

During the final three days of the study, five hens from each treatment had their fresh fecal samples taken every 24 h. Manure and feed intake were measured; then the fecal samples were dried at 65ºC to a consistent weight and stored for laboratory examination (dry matter, organic matter, crude protein and ether extract). The nutrient retention percent was calculated according to A.O.A.C (2005) the following equation:

$$Nutrient\;retention\;\%=(nutrient\;intake-nutrient\;excretion)\ast100/nutrient\;intake$$

### Blood biochemical profiles

On the final day of the experiment, five hens were randomly selected from each treatment group to draw blood from the brachial wing vein of the living hens (without giving them anesthesia or euthanizing them). Then, the blood samples with heparin substance were immediately centrifuged at 3000 rpm for 20 min, and the plasma was stored at −20ºC for further analysis. The following blood parameters were measured: total protein, albumin, globulin, cholesterol, triglycerides, alanine transaminase (ALT), aspartate aminotransferase (AST), and estradiol hormone (E2). Except for estradiol hormone (E2), which was determined by immunoassay analyzer using “iFlash kits”, all parameters were determined colorimetrically using “BioMed diagnostic kits”. Subtracting albumin from total protein yielded globulin.

### Data analysis

The data was analyzed by using a simple one-way analysis of variance (ANOVA), SAS program (2002) in accordance with this model:

$$\mathrm{Yij}=\mathrm\mu+\mathrm{Ti}+{\;\mathrm{eij}},$$

where: Yij = Observation, µ = General mean, Ti = Random effect of treatment (i = 1, 2, 3 and 4), eij = Random error. The separation of means took place by Duncan test (Duncan 1955) with level of significance is *P* < 0.05.

## Results

### Laying hens' performance and productivity

While the improvement from treating with formic and propionic acids compared to the control was not statistically significant, treating with acids mixture significantly (*P* < 0.05) increased both egg mass and the percentage of hen-day egg production. There was no significant difference observed among the treatments in terms of egg weight or feed intake. When comparing the experimental groups to the control, the feed conversion ratio showed a numerical improvement (Table 2).

**Table 2:** The effect of formic acid, propionic acid, and their mixture on the production performance of laying hens during the early production phase

Item	Treatments				SE	*P* value
	C	F	P	M		
Hen-day egg production%	67.65^b^	70.80^b^	70.41^b^	81.56^a^	3.516	0.0486
Egg mass (g)	42.90^b^	44.00^b^	44.25^b^	51.19^a^	2.895	0.0540
Egg weight (g)	63.39	62.15	62.82	62.83	0.619	0.5765
Feed intake (g/hen/day)	109.66	103.41	95.9	102.16	7.684	0.6602
Feed conversion ratio (FI/Emass)	2.55	2.35	2.16	1.99	0.171	0.1686

^a,^^b^Means within the same row showing different letters are significantly different at *P*<0.05

*SE* standard error, *Emass* Egg mass

### Egg and shell quality characteristics

The impact of experimental treatments on egg and shell quality traits is shown in Table 3. The experimental treatments had no effect on egg weight, egg shape index, shell weight, shell%, thickness, surface area, or SWUSA. In comparison to the other groups, the formic acid group increased the percentages of shell Ca (*P* < 0.001), yolk (*P* < 0.01), and decreased albumen percent (*P* < 0.01). The experimental treatments resulted in a significant increase (*P* < 0.05) in Haugh unit and albumen index when compared to the untreated group. The highest values of shell density (*P* < 0.05) were observed with the propionic acid group, followed by the mixture group.

**Table 3:** Effect of formic acid, propionic acid, and their mixture on egg and shell characteristics

Item	Treatments				SE	*P* value
	C	F	P	M		
Egg weight (g)	63.96	64.58	65.46	63.46	2.177	0.9199
Egg shape index	80.56	78.88	81.81	81.66	1.207	0.2620
Albumen %	61.16^a^	57.59^b^	61.16^a^	60.94^a^	0.897	0.0149
Yolk %	26.07^b^	30.02^a^	26.17^b^	27.19^b^	0.930	0.0148
Haugh unit	83.51^b^	87.61^a^	89.05^a^	87.90^a^	1.22	0.0274
Albumen index	9.93^b^	11.72^a^	11.71^a^	11.08^ab^	0.477	0.0543
Yolk index	40.89^b^	43.83^ab^	49.66^a^	46.05^ab^	2.049	0.0491
Shell weight (g)	7.400	7.15	7.39	6.71	0.373	0.5141
Shell %	11.60	11.03	11.31	10.65	0.536	0.6562
Shell thickness (mm)	0.553	0.507	0.463	0.465	0.027	0.1100
Shell Ca%	42.06^b^	47.92^a^	34.23^c^	37.76^c^	1.095	0.0001
Shell surface area (cm^2^)	74.75	75.23	76.02	74.31	1.794	0.9131
SWUSA (mg/cm^2^)	99.01	94.59	97.29	90.57	4.499	0.5806
Shell density (g/cm^3^)	0.180^b^	0.189^b^	0.211^a^	0.195^ab^	0.006	0.0180

^a,b,c^Means within the same row showing different letters are significantly different at *P* < 0.05 and *P* < 0.01

*SE* Standard error

### The chemical composition of egg yolk and albumen

Table 4 displays the impact of the experimental treatments on the percent of fat and crude protein (CP) of the egg yolk and albumen. When the mixture of acids was applied, the low values of albumen fat percent (*P* < 0.05) and yolk fat percent (*P* < 0.001) were noted. Compared to the other treatments, the albumen fat percentage was significantly higher (*P* < 0.05) in the propionic acid treatment, followed by the formic acid treatment. On the other hand, there was a significant increase (*P* = 0.0007) in the CP of albumen with the acids’ mixture treatment, while the differences among the treatments were not statistically significant at the CP of yolk.

**Table 4:** Effect of formic acid, propionic acid, and their mixture on the composition of egg yolk and albumen (On dry matter basis)

Item	Treatments				SE	*P* value
	C	F	P	M		
Fat%						
Yolk	62.30^a^	63.24^a^	62.83^a^	56.62^b^	0.720	0.0006
Albumen	1.98^b^	4.09^ab^	6.32^a^	1.70^b^	0.919	0.0245
CP%						
Yolk	21.81	19.04	19.55	21.70	2.344	0.7735
Albumen	39.64^b^	44.81^b^	47.71^b^	71.65^a^	3.383	0.0007

^a,b^Means within the same row showing different letters are significantly different at *P* < 0.05 and *P* < 0.001

*SE* Standard error

### Digestibility trial

Table 5 shows the retention percentages for dry matter (DM), organic matter (OM), crude protein (CP), and ether extract (EE). The experimental treatments had no significant effect on the retention of DM and OM, while they significantly (*P* < 0.01) increased the retention of CP when compared to the untreated group. When comparing the experimental treatments to the control, there was a tendency for ether extract retention to rise (*P* = 0.1) in treatment groups.

**Table 5:** Effect of formic acid, propionic acid, and their mixture on nutrient retention

Item	Treatments				SE	*P* value
Retention%	C	F	P	M		
DM	75.06	81.09	73.18	76.52	3.502	0.4264
OM	76.27	82.47	75.42	79.17	3.292	0.4231
CP	70.53^b^	89.33^a^	87.26^a^	88.09^a^	2.636	0.0029
EE	74.16	86.26	77.85	80.48	6.766	0.1197

^a,b^Means within the same row showing different letters are significantly different at *P* < 0.01

*SE* Standard error

### Blood biochemical profiles

Table 6 shows the influence of the experimental treatments on plasma cholesterol, triglycerides, alanine transaminase (ALT), aspartate aminotransferase (AST), total protein, albumin, globulin, and estradiol hormone (E2). The experimental treatments resulted in a significant (*P* < 0.001) decrease in plasma triglycerides when compared to the untreated group. In terms of plasma cholesterol, the acids’ mixture group had the lowest value (*P* < 0.001), followed by the formic acid group, but the propionic acid group had the highest value (*P* < 0.001). The differences in plasma ALT, AST, and albumin levels among treatments were not statistically significant. When contrasted with the other treatments, the acids’ mixture treatment significantly reduced plasma total protein and globulin (*P* < 0.05). The highest level of estradiol hormone (E2) (*P* < 0.001) was observed with the acids’ mixture treatment, while the lowest level was noticed with the formic acid group.

**Table 6:** Effect of formic acid, propionic acid, and their mixture on some blood metabolites

Item	Treatments				SE	*P* value
	C	F	P	M		
Cholesterol (mg/dl)	119.35^b^	88.45^c^	164.00^a^	78.65^d^	2.026	0.0001
Triglycerides (mg/dl)	661.23^a^	480.90^b^	324.60^c^	404.6^bc^	34.186	0.0007
ALT (u/l)	23.33	14.00	21.00	27.33	5.742	0.4617
AST (u/l)	201.00	265.00	308.33	256.33	42.467	0.4116
Total protein (g/dl)	9.10^a^	9.50^a^	9.63^a^	7.30^b^	0.499	0.0358
Albumin (g/dl)	1.40	1.50	1.53	1.53	0.103	0.7721
Globulin (g/dl)	7.70^a^	8.00^a^	8.10^a^	5.77^c^	0.440	0.0167
Estradiol hormone (pg/ml)	249.33^b^	87.07^d^	215.67^c^	313.00^a^	5.006	0.0001

^a,b,c,d^Means within the same row showing different letters are significantly different at *P* < 0.05 and *P* < 0.001

*SE* Standard error

## Discussion

The modern poultry industry requires high levels of production and efficient feed conversion, which can be partially met by using certain feed additives like organic acids, which act as growth promoters to increase hen productivity and inhibit gut pathogens. The combination between formic and propionic acids may be responsible for the improvement in egg productivity and egg mass resulting from the application of acids mixture treatment. Specifically, they reduce the pH of the crop and gizzard, preventing the colonization of pathogens on the gut wall and enhancing feed efficiency, total nutrient digestibility, and egg laying rate (Wang et al. 2009). These results are consistent with a number of studies that found that feeding laying hens a combination of formic and propionic acids in their diets considerably boosted their egg production and egg mass much more than the control (Grashorn et al. 2013; Dahiya et al. 2016; Salem 2020). Organic acid treatment (3g/kg diet) was shown to have no substantial effect on feed intake, or feed conversion ratio (Shalaei et al. 2014). Contrary to the current results, the addition of dietary organic acids did not significantly affect egg production (Shalaei et al. 2014) or egg mass (Boling et al. 2000). The feed intake, feed conversion ratio, and egg weight in female Japanese quails were dramatically raised by diets containing lactic acid, butyric acid, and acetic acid combined, as well as acetic acid alone (Fouladi et al. 2018). The rise in shell Ca% caused by formic acid addition to the feed of laying hens was most likely due to a decrease in intestinal pH, which produces an increase in calcium solubility, availability, and absorption (Van den Heuvel et al. 1999). This increase was seen as an excellent measure of shell quality and could be attributed to formic acid's beneficial effect on the intestinal absorption rate of Ca, which enhanced shell Ca deposits. The Haugh unit is a strong indicator of egg protein quality and freshness (Rath et al. 2015). The significant increase in albumen index and Haugh unit as a result of organic acid treatments is an indication of the high quality of the eggs produced. The United States Department of Agriculture (USDA) considers eggs AA (72 or higher) to be of excellent quality based on Haugh unit (Haugh 1937). The current findings are consistent with a prior study, which found that an organic mixture combining propionic acid and formic acid produced higher shell quality ratings (Park et al. 2009). Additionally, it has been noted that the egg shape index and shell weight of laying hens were not significantly affected by dietary organic acids or their combination (Sari and Kaya 2017). The feeding of diets containing organic acids to hens improved their Haugh unit score (Wang et al. 2009). In addition, meals containing 0.01% or 0.15% formic acid enhanced laying hens’ Haugh unit score (Abbas et al. 2013). The rise in albumen CP% was most likely due to increase in digestive tract acidity caused by feeding an organic acid combination to hens, which converted pepsinogen into pepsin, increasing protein and amino acid absorption (Park et al. 2009). However, the use of a mixture of formic and propionic acids resulted in a drop in yolk and albumen fat percentage, which is congruent with the current study's reduction in plasma cholesterol and is thought to be an indication of low yolk cholesterol (Aghaii et al. 2010). These results contradict previous findings that utilizing formic, propionic, and their combination in laying hens’ diets resulted in non-significant variations in egg fat and protein% among treatments in the final production stage (Salem 2020). Organic acids' tendency to lower the acidity of chickens' gut (Leeson et al. 2005) improves pepsin activity, which promotes protein digestibility (Afsharmanesh and Porreza 2005). Furthermore, organic acids stimulate gastric proteolysis as well as protein and amino acid digestion (Samanta et al. 2010). Additionally, organic acids promote pancreatic juice synthesis, which may aid protein digestion due to the presence of high amounts of trypsinogen, chymotrypsinogen A, chymotrypsinogen B, pro-carboxy peptidase A, and pro carboxy peptidase B (Adil et al. 2010). The present findings corroborate those of previous studies that demonstrated the presence of ascorbic and citric acids in lime juice enhanced the nutritional digestibility of broiler chickens (Ndelekwute and Enyenihi 2017). Likewise, the digestibility of crude protein and ether extract was markedly enhanced by all of the diets that contained various organic acids (acetic, butyric, citric, and formic) (Ndelekwute et al. 2019). In addition, 30 g fumaric acid/kg diet considerably enhanced the apparent digestibility of crude protein and ether extract versus the untreated group, while 30 g citric acid/kg diet exhibited a non-significant increase in CP digestibility (Attia et al. 2018). Furthermore, 5 g fumaric or 5 ml formic acid and 7.5 ml acetic or 20 g citric acid/kg diet increased broiler diet CP and EE digestibility (Ghazalah et al. 2011). Additionally, 20 g citric acid/kg diet improved CP retention in broiler diet (Ao et al. 2009). The digestibility of EE and CP was considerably higher in the ascorbic acid group than in the untreated group (Lohakare et al. 2005). Organic acids have the potential to lower plasma cholesterol levels by stimulating the synthesis of bile acid (Imaizumi et al. 1992) and decreasing the synthesis of hepatic cholesterol (Hara et al. 1999). This finding is consistent with other earlier research showing that adding formic acid, either alone or in combination with propionic acid, to the diets of laying hens significantly reduced their plasma cholesterol levels (Salem 2020). Dietary acidifiers dramatically reduced blood cholesterol in broilers (Kamal and Naela 2014). Furthermore, the serum triglyceride content of the female Japanese quail groups given either acetic acid or lactic acid was dramatically reduced, while total protein, albumin, globulin, and cholesterol levels rose (Fouladi et al. 2018). The action of organic acids with the increase in protein synthesis, which boosted the synthesis of lipoprotein in liver cells and transported the triglycerides from the blood into the liver cells, may be responsible for the reduction in plasma triglycerides in the present study (Adil et al. 2010). ALT and AST are biochemical indicators of liver health and function (Che et al. 2011). Treatments with organic acids resulted in non-significant variations in plasma ALT, AST, and albumen, whereas formic acid and its combination with propionic acid demonstrated a substantial drop in plasma cholesterol (Salem 2020). Organic acids in laying meals (Sari and Kaya 2017), acetic acid at different ages of Japanese quail (Attia et al. 2013), and acetic, citric, and lactic acids in broiler diets (Abdel-Fattah et al. 2008) had no effect on ALT and AST enzymes. Conversely, the organic acids mixture markedly boosted AST activity in laying diets (Yesilbag and Ҫolpan 2006). One major reproductive hormone that influences sexual differentiation and behavior in addition to the growth, development, maturation, and functioning of the reproductive tracts is estradiol hormone (E2) (Balthazart et al. 2009). Moreover, E2 starts mRNA expression and albumen protein synthesis from the oviduct (Salomaa et al. 1992). Similarly, it influences hepatic function and activates precursors of egg yolks (Dashti et al. 1983). Unfortunately, no scientific studies have been published on the effect of organic acids on the estradiol hormone. However, a recent study concluded that the combination of probiotics (which has the potential to improve the intestinal fermentation rate and generation of short-chain fatty acids (Alagawany et al. 2018) and prebiotics increased estradiol hormone greatly compared to the control (Salem and Abd El-Dayem 2024).

## Conclusions

The combination of formic acid (0.5 ml/kg diet) and propionic acid (0.5 ml/kg diet), when added to the diets of laying hens at the beginning of their egg production, was found to have a positive effect on both egg production and the quality of the egg and shell. Additionally, this combination of acids showed the lowest percent of egg albumen fat and the highest percent of albumen crude protein. Furthermore, neither the nutritional value of the eggs produced nor the overall health of the hens is negatively impacted by these feed additives. As a result, we recommend that laying hens' diets during the early stage of production should contain a blend of propionic and formic acids.

## Data Availability

All data generated and analyzed during this study are included in this published article.
